# Fever of unknown origin revealing testicular nocardiosis: a case report and literature review

**DOI:** 10.1186/s12879-024-09521-8

**Published:** 2024-06-21

**Authors:** Saohoine Inthasot, Sophie Leemans, Mony Hing, Julien Vanderhulst

**Affiliations:** 1 Department of Internal Medicine, CHU Brugmann, Université Libre de Bruxelles, Brussels, Belgium; 2 Department of Infectious Diseases, CHU Brugmann, Université Libre de Bruxelles, Brussels, Belgium; 3 Department of Microbiology, Laboratoire Hospitalier Universitaire de Bruxelles-Universitair Laboratorium Brussel (LHUB-ULB), Université Libre de Bruxelles, Brussels, Belgium

**Keywords:** *Nocardia*, Epididymo-orchitis, Scrotal abscess, Testicular nocardiosis, Brain abscess, Disseminated nocardiosis, Fever of unknown origin

## Abstract

**Background:**

*Nocardia* is an ubiquitous soil organism. As an opportunistic pathogen, inhalation and skin inoculation are the most common routes of infection. Lungs and skin are the most frequent sites of nocardiosis. Testis is a highly unusual location for nocardiosis.

**Case presentation:**

We report the case of an immunocompromised 75-year-old-man admitted for fever of unknown origin. He presented with skin lesions after gardening and was first suspected of Mediterranean spotted fever, but he did not respond to doxycycline. Then, physical examination revealed new left scrotal swelling that was compatible with a diagnosis of epididymo-orchitis. The patient’s condition did not improve despite empirical antibiotic treatment with the onset of necrotic scrotal abscesses requiring surgery. *Nocardia brasiliensis* yielded from the removed testis culture. High-dose trimethoprim-sulfamethoxazole and ceftriaxone were started. Multiple micro-abscesses were found in the brain and spinal cord on imaging studies. After 6 weeks of dual antibiotic therapy for disseminated nocardiosis, slight regression of the brain abscesses was observed. The patient was discharged after a 6-month course of antibiotics and remained relapse-free at that time of writing these lines. Trimethoprim-sulfamethoxazole alone is meant to be pursued for 6 months thereafter. We undertook a literature review on previously reported cases of genitourinary and urological nocardiosis; to date, only 36 cases have been published with predominately involvement of kidney, prostate and testis.

**Conclusions:**

To the best of our knowledge, this is the first case of *Nocardia brasiliensis* simultaneously infecting skin, testis, brain and spinal cord in an immunocompromised patient. Knowledge on uncommon forms of nocardiosis remains scarce. This case report highlights the difficulty of diagnosing atypical nocardiosis and the importance of prompt bacteriological sampling in case of empirical antibiotics failure.

## Background

Members of the genus *Nocardia* are aerobic, Gram-positive, beaded, and partially acid-fast bacilli with the microscopic appearance of branching hyphae, belonging to the *Corynebacterineae* suborder. They are ubiquitous soil organisms. As an opportunistic pathogen, inhalation and skin inoculation are the most common routes of infection. Lungs and skin are the most frequent sites of nocardiosis. Testis is a highly unusual location for nocardiosis. We herein report the case of an immunocompromised patient with fever of unknown origin unmasking disseminated nocardiosis involving testis, brain and spinal cord. We have included a literature review on previous case reports of genitourinary and urological nocardiosis.

## Case presentation

A 75-year-old man was admitted for fever of unknown origin. He had previously been diagnosed with polymyalgia rheumatica, for which a treatment with methylprednisolone 16 mg once a day (OD) was begun 4 months before admission. Methotrexate 10 mg weekly had been introduced 2 months before his admission. He had a past history of acquired amegacaryocytic thrombocytopenia that had been treated with cyclosporin more than 10 years ago.

After his two-month vacation in South of France, where he had been gardening without wearing gloves, he developed a fever above 39 °C with complaints of sore throat. Amoxicillin-clavulanic acid was started after the patient was seen in the emergency room of another hospital.

**Fig. 1:**
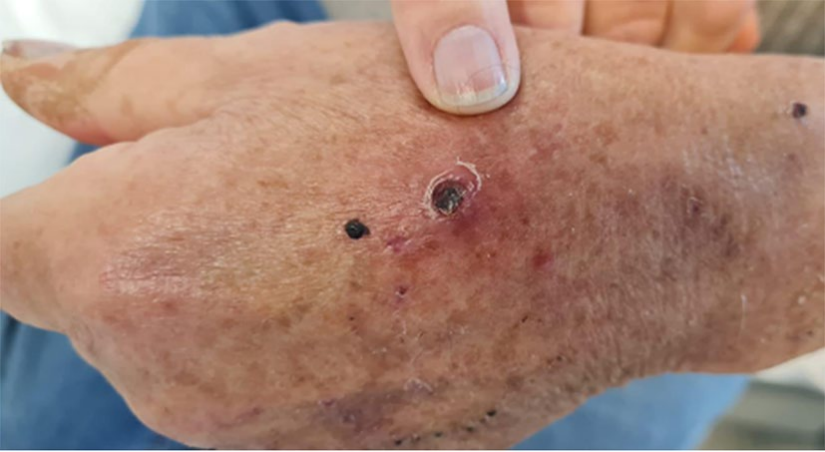
Skin necrotic lesion on the back of the hand

As the fever persisted, he presented to the emergency room of our institution. An atypical papular skin rash with a necrotic lesion on the back of the left hand, combined with his recent vacation location, prompted an initial suspicion of Mediterranean spotted fever (Fig. 1).

Therefore, doxycycline 100 mg BID was introduced as an empirical treatment. Antibiotics were continued for 7 days. Meanwhile, a first serologic test for *Rickettsia conorii* turned negative. Fever and malaise persisted for more than two weeks in total and, after a week of ineffective doxycycline, antibiotics were discontinued.

After 2 weeks of pyrexia, the patient was hospitalized in our internal medicine department. He initially complained primarily about fatigue. He had no arthralgia. Nobody close to him was sick and there was no history of animal exposure. On admission, his vital signs were as follows: body temperature 37,8 °C; pulse rate 66/min; and blood pressure 110/60 mmHg. A physical examination revealed virtual disappearance of the skin lesions and a new aortic heart murmur. First-line laboratory analyses showed an elevation of the C-reactive protein level (CRP) at 45,0 mg/L (normal value < 5,0 mg/L). Transesophageal echocardiography did not show any evidence of infective endocarditis. A chest, abdomen and brain computed tomography (CT) was unremarkable. Methotrexate was discontinued on admission.

No obvious sign of vasculitis was noted on CT brain angiography. Serologic testing for *Brucella, Rickettsia conorii and R. mooseri, Coxiella burnetii, Bartonella henselae*, *Borrelia burgdorferi*, *Treponema pallidum* and human immunodeficiency virus proved negative. Cytomegalovirus and Epstein-Barr virus serologies were compatible with past infection. Rheumatoid factor and antinuclear antibodies turned negative. Repeated blood cultures remained sterile even after prolonged incubation of 7 days.

After 3 weeks of unexplained intermittent pyrexia, the patient was diagnosed with fever of unknown origin. Then, he mentioned a new scrotal swelling and left epididymo-orchitis was confirmed by ultrasound. Urinalysis was normal with no pyuria. Levofloxacin 500 mg OD was started empirically. In spite of a decrease in pain, swelling and CRP level, testis induration persisted. Scrotal abscesses appeared after one week of antibiotic therapy and despite increased levofloxacin doses, they evolved to necrosis. Left orchiectomy was performed. Clindamycin 600 mg TDS and ceftriaxone 2 g OD were started empirically. The dose of methylprednisolone was progressively reduced to a nadir of 2 mg OD.

**Fig. 2:**
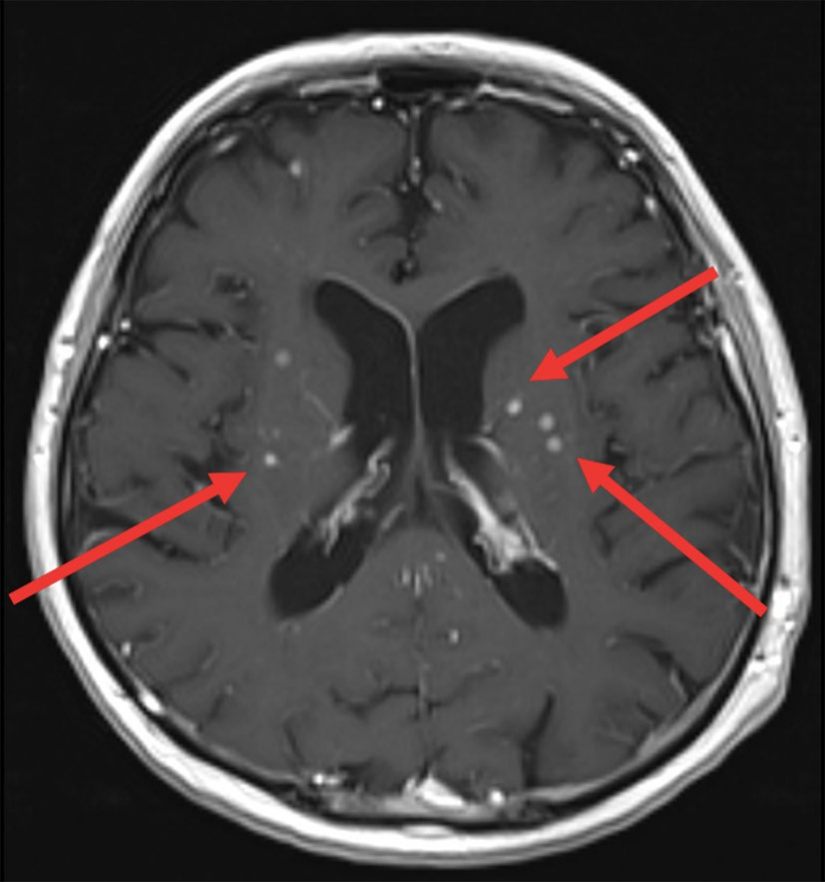
MRI of brain T1 (+ gadolinium) sequence, showing multiple micro-abscesses (hyperintensities pointed by the arrows)

**Fig. 3:**
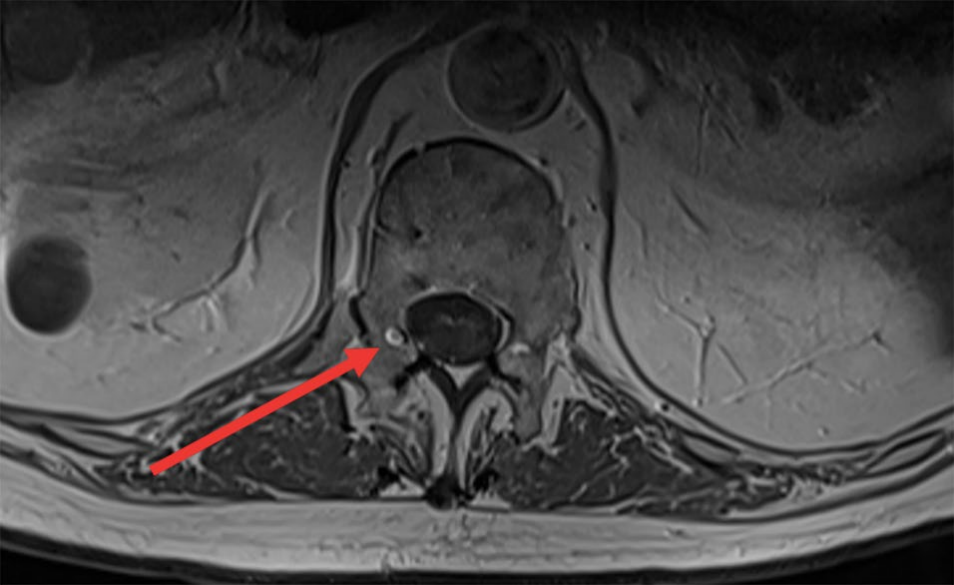
MRI of spinal cord T1 (+ gadolinium) sequence, showing a 2-mm abscess with a “ring enhancement” in right posterolateral area of the spinal cord in D12-L1 (arrow)

*Nocardia brasiliensis* yielded from the testicular biopsy culture. High dose intravenous trimethoprim-sulfamethoxazole (TMP-SMX) (20 mg TMP/kg/day) was started on the 20th day of hospitalization. Ceftriaxone was increased to 2 g BID to treat potential brain involvement. Gadolinium contrast-enhanced magnetic resonance imaging (MRI) of the brain subsequently revealed multiple micro-abscesses mostly in the nucleus nuclei, dura-mater enhancement in the spinal bulb and ventriculitis (Fig. 2). Spinal cord MRI showed a “ring-enhancement” in right posterolateral area of the spinal cord in D12-L1, which was consistent with a 2 mm-abscess (Fig. 3).

A F-18 fluorodeoxyglucose positron emission tomography/CT was performed to assess the extent of invasive nocardiosis but was unremarkable.

Multi-susceptibility was confirmed and dual parenteral antibiotic therapy with TMP-SMX and ceftriaxone was pursued for 6 weeks.

**Fig. 4:**
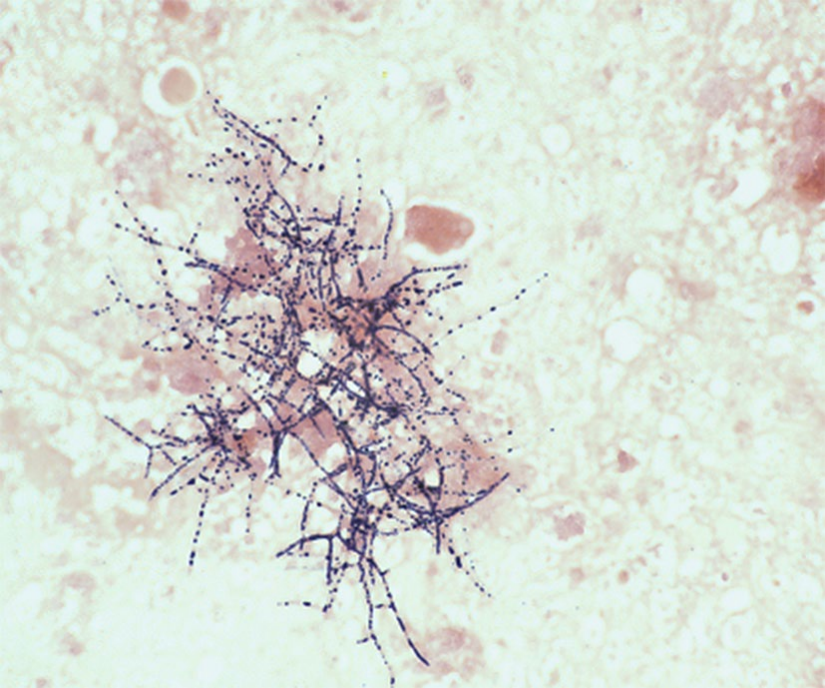
Microscopic image of a gram-stained smear of *N. brasiliensis* from testicular biopsy, demonstrating the typical gram-positive filamentous bacilli. Magnification, x1000

Histopathologically, signs of inflammation were observed in the testicular biopsy, as well as filamentous branching bacilli. Direct Gram staining showed the typical gram-positive, beaded, filamentous bacilli (Fig. 4).

**Fig. 5:**
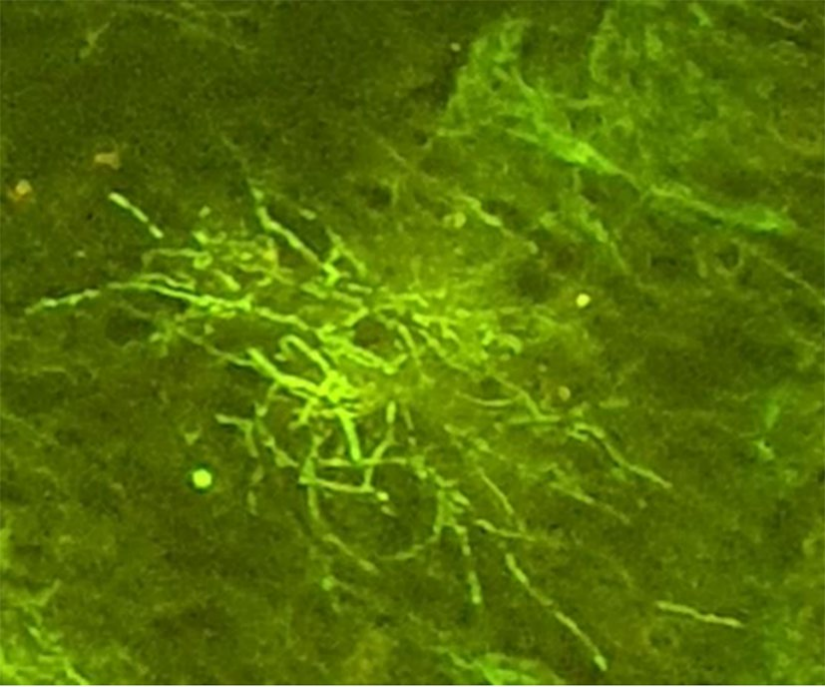
Microscopic image of auramine-rhodamine modified stained smear of *N. brasiliensis* from testicular biopsy. Magnification, x1000

The colonies displayed also partial acid-fastness with the modified form of auramine-rhodamine stain (Fig. 5).

**Fig. 6:**
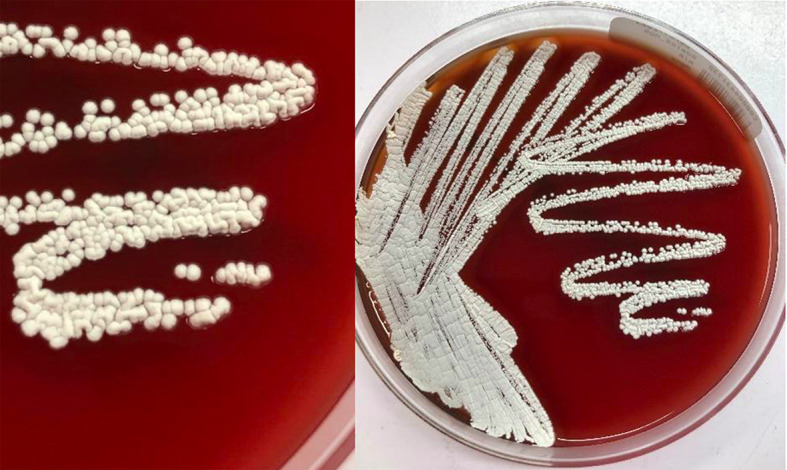
Colonies of *N. brasiliensis* grown on blood agar plate

After 72 h incubation in 5% CO_2_ at 37 °C on sheep blood agar, colonies grew, appearing as chalky white cotton balls because of the presence of abundant aerial filaments (Fig. 6).

*Nocardia brasiliensis* was identified by matrix-associated laser desorption ionization-time of flight mass spectrometry (MALDI-TOF Biotyper Sirius IVD version 4.2.100; Bruker Daltonics, Bremen, Germany) with a reliable score value (2.06). To confirm the germ identification, 16S rRNA gene sequencing using universal primers (27F: 5’AGAGTTTGATCMTGGCTCAG3’ and 1492R: 5’TACGGYTACCTTGTTACGACTT3’; NF1: 5’TWACACATGCAAGTCGARCG3’ and NF2: 5’CCAACATCTCACGACACGAG3’) was performed on cultured colonies. Its yielded sequence (1025 bp) had 99.90% homology with *N. brasiliensis* strain DSM AUSMDU00024985 (GenBank accession no.: CP046171.1) by using the NCBI database and the EZBiocloud.

The antimicrobial susceptibility testing was performed by minimum inhibitory concentration (MIC) using E-test gradient strips (BioMérieux, Marcy l’Etoile, France) and interpreted following Clinical and Laboratory Standards Institute (CLSI) M62-ED1:2018 guidelines for *Nocardia* (Table 1).

**Table 1:** Antimicrobial susceptibility of patient’s clinical isolate *N. brasiliensis*

Antibiotics	MIC (µg/mL)	Interpretation by CLSI M62 guidelines
Amoxicillin-clavulanic acid	0.500	S
Ceftriaxone	1.000	S
Meropenem	2.000	S
Clindamycin	0.380	S
Moxifloxacin	0.125	S
TMP-SMX	0.125	S
Linezolid	1.000	S

## Outcome

**Fig. 7:**
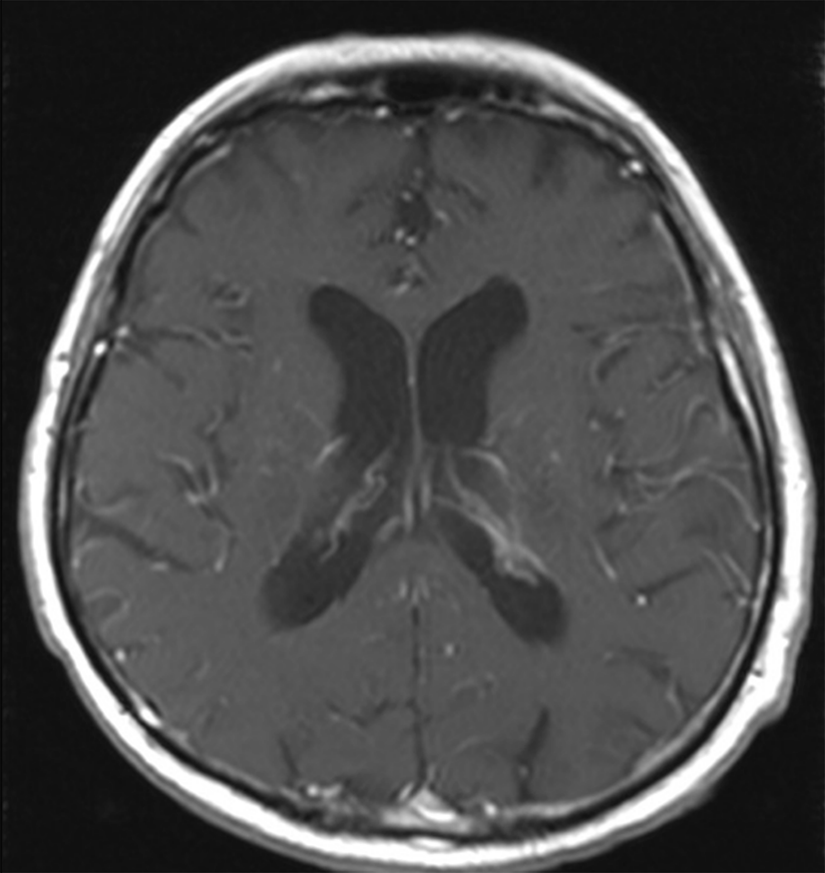
RMI of brain T1 (+ gadolinium) sequence, performed after 6 weeks of antibiotics, showing slight regression of the abscesses

**Fig. 8:**
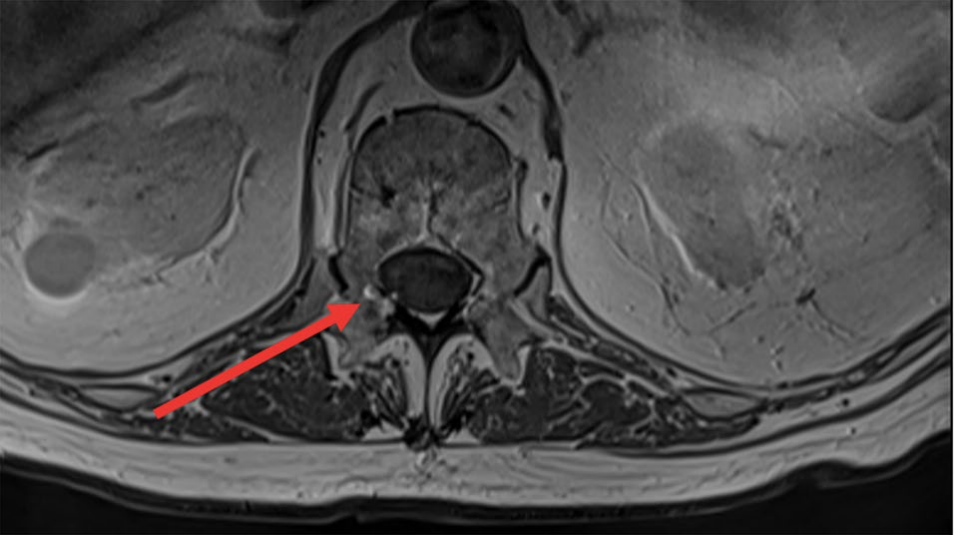
RMI of spinal cord T1(+ gadolinium) sequence, performed after 6 weeks of antibiotics, showing a stable abscess (arrow)

Clinical evolution was marked by a slow improvement of the patient’s general condition. Fever gradually decreased in intensity and frequency. Control brain and spinal cord MRI’s were obtained shortly before the end of the 6 weeks of parenteral bitherapy (Figs. 7 and 8): a slight regression of the brain micro-abscesses was observed, and they appeared to be less enhanced by gadolinium, while the spinal cord lesion remained stable.

High dose TMP-SMX was then switched from parenteral to oral route and pursued as a monotherapy. At the time of writing these lines, the patient has just been discharged from the rehabilitation department after almost 6 months of treatment. Another control brain MRI showed further reduction of the abscesses. Clinically, the patient still has some walking impairment that requires physiotherapy, with slow but constant improvement. TMP-SMX is meant to be pursued for 6 months thereafter.

## Discussion

Nocardiosis most commonly affects immunocompromised patients but may occur in immunocompetent hosts [1–4]. Disseminated nocardiosis is defined as the involvement of at least two non-contiguous organs and/or demonstration of bloodstream infection [1, 2, 4, 5]. The most frequently infected sites are the lungs, brain and skin [3, 6, 7]. Fever at presentation is inconstant [1–4]. Diagnosing nocardiosis only by skin inspection is difficult because *Nocardia* lesions are not specific. They may appear as papules, pustules, nodules and skin infiltration [1, 5, 6]. In our case, with the patient’s epidemiological context, the skin lesions were considered suspicious of a rickettsial disease at the time of presentation. *Nocardia* skin inoculation while gardening seems to be the pathway to infection in our patient.

*Nocardia* infects the central nervous system (CNS) in one-third of all cases and it usually manifests as brain abscess while meningitis is rare [5, 8]. Multiple abscesses are seen in 50–80% of the patients [6]. It has been very rarely reported with *N. brasiliensis*. Patients sometimes present with headache, nausea, vomiting, seizure or alteration in consciousness [1, 7]. CNS invasion may nevertheless be asymptomatic and missing the diagnosis of CNS nocardiosis may cause treatment delay and failure. It has been suggested to perform systematic brain MRI to all patients with a diagnosis of nocardiosis [5, 6]. In our case, lumbar puncture was discussed but not performed because of thrombocytopenia (less than 50 000 platelets/µL), reactions to previous platelet transfusions and clinical improvement with antibiotics. Besides, *Nocardia* is only exceptionally identified in cerebral spinal fluid culture [8]. Although invasive nocardiosis is generally considered to occur through hematogenous dissemination, identifying *Nocardia* species on blood culture is very uncommon [2, 5].

Infections of the urinary and urological systems are usually caused by species of the family *Enterobacteriaceae*; *Nocardia* infection is extremely rare [4, 8, 9]. We searched PubMed for English-language reports of genitourinary and urological nocardiosis from 1970 to 2022. We found 36 complete cases previously published in the literature [9–41] (Table 2). All except four cases (nr 17, 27, 29 and 31) involved immunocompromised hosts, mostly transplant patients and patients on corticosteroids. Kidneys, prostate and testes were the most commonly infected organs. *N. asteroides* was the most frequent pathogen. Implication of *N. brasiliensis* causing urological and genitourinary infection seems rarer but all cases reported skin involvement. It has been described that this strain is more prevalent in cutaneous infections [1, 9]. In our case, *N. brasiliensis* is thought to have spread hematogenously from the skin to testicular, cerebral and spinal cord sites.

**Table 2:** Cases of genitourinary and urological nocardiosis reported in the literature

Nr	Year	Author (reference)	Nocardia species	Age (years), gender	Underlying conditions	Immunosuppressants	Clinical presentation	Nocardia identification specimen type	Sites of Nocardia infection	Diagnosis delay	Antibiotic empirical treatment	Antibiotic targeted treatment (+ duration if specified)	Surgical management	Outcome + follow-up
1	1964	Mahvi et al. [10]	*N. brasiliensis*	32, M	Multiple myeloma	Corticosteroids	Anorexia Weight loss Abdominal pain	Renal abscess Skin abscess (autopsy)	Kidney Skin Blood Lungs	2 months	penicillin	sulfonamide	None	Deceased 20 days after admission
2	1971	Young et al. [11]	*N. asteroides*	53, M	Hodgkin’s lymphoma	Alkylating agents Corticosteroids Vinca alkaloid	Hemoptysis Weight loss Fever	Sputum Scalp abscess Renal and cerebellar abscesses (autopsy)	Lung Brain Kidney	2 days	isoniazide + streptomycin	sulfonamide	None	Deceased 3 days after targeted treatment
3	1971	Young et al. [11]	*N. asteroides*	26, M	Hodgkin’s lymphoma	NR	Fever	Sputum Renal abscess Cerebral abscess (autopsy)	Lung Kidney Brain	NR	NR	NR	None	Deceased 3 days after targeted treatment
4	1971	Young et al. [11]	*N. asteroides*	41, F	Lymphosarcoma	NR	Fever	Sputum Renal abscess (autopsy)	Lung Kidney	NR	methicillin + chloramphenicol Then cephalothin	NR	None	Deceased 6 days after targeted treatment
5	1971	Young et al. [11]	*N. asteroides*	44, M	Chronic myelogenous leukemia	Alkylating agent Corticosteroids	Oral + pharyngeal ulcers Recurrence with adenopathy, fever and scrotal swelling	Oral scraping Sputum Testicular abscess (autopsy)	Mouth Pharynx Lung Testis	3 weeks	None	sulfonamide	None	Deceased 6 weeks after admission
6	1973	Diamond et al. [12].	*N. brasiliensis*	65, M	Systemic lupus erythematosus Diabetes mellitus	Corticosteroids	Fever Nodular skin lesions Scrotal swelling Recurrence 1 year later	Skin lesion Urine Urine	Skin Prostate Prostate	NR	None	sulfonamide Then tetracycline (2 months) sulfonamide (12 months)	None	No recurrence 1 year after targeted treatment Deceased 1 year after remission
7	1974	Geelhoed et al. [13]	*N. asteroides*	70, M	Waldenström’s macroglobulinemia Lung adenocarcinoma	Corticosteroids	Hemoptysis Scrotal swelling	Testicular abscess Sputum	Testis Lung	6 weeks	chloramphenicol + tetracycline Then sulfonamide	sulfonamide	Orchiectomy	Deceased
8	1976	Strong et al. [14]	*N. asteroides*	61, M	Myeloproliferative disorder Splenectomy	Corticosteroids Alkylating agents	Persistent urinary infection Fever	Testicular abscess BAL fluid	Testis Prostate Lungs Liver	NR	cephalothin + gentamicin	+ carbenicillin + sulfadiazine + ampicillin + cycloserine	Orchiectomy	Deceased 17 days after admission
9	1986	Wheeler et al. [15]	*N. asteroides*	44, M	Heart Tx	Corticosteroids Azathioprine Cyclosporin	Scrotal swelling Decreasing vision	Testicular abscess	Testis Eye	NR	tetracycline	sulfonamide	Orchiectomy	Survived 3 months after follow-up
10	1992	Parmentier et al. [16]	*N. farcinica*	36, M	HIV infection	NR	Fever Loin pain	Renal abscess Sputum	Kidney Lungs	NR	None	imipenem + rifampin (2 months)	Nephrectomy	Survived 4 months after follow-up
11	1994	Shohaib S [17]	*N. asteroides*	45, F	Kidney Tx	Cyclosporin Corticosteroids Azathioprine	Abdominal pain Fever	Perinephric abscess	Kidney Iliopsoas muscle	NR	ciprofloxacin + cefuroxime	ciprofloxacin + cefuroxime (more than 1 month)	None	Survived 1 year after follow-up
12	1994	Lopez et al. [18]	*N. asteroides*	52, M	Liver Tx	Cyclosporin Corticosteroids Azathioprine	Scrotal swelling Subcutaneous abscess	Skin abscess	Subcutaneous tissue Testis	NR	None	TMP-SMX (9 months)	Orchiectomy	Survived 2 years after follow-up
13	1994	Miralles et al. [19]	*N. farcinica*	24, M	HIV infection	NR	Fever Weight loss Cough Recurrence with headache, hemiparesis	Renal abscess BAL fluid CSF	Kidneys Lungs Brain	NR	cefixime Then TMP-SMX + zidovudine	TMP-SMX Switch doxycyclin + ciprofloxacin TMP-SMX	None	Survived
14	1996	Salahuddin et al. [20]	*N. asteroides*	47, F	Type 1 diabetes	None	Dysuria Loin pain Fever	Urine Blood	Kidney Blood	NR	NR	sulfonamide	None	Survived
15	2000	Torres et al. [40]	*N. farcinica*	85, M	Non-Hodgkin lymphoma COPD	Alkylating agent Corticosteroids	Fever Cough Malaise Fatigue	Urine Blood	Kidney Blood Lung	15 days	cefotaxime + indomethacin	cefotaxime + indomethacin	None	Deceased 20 days after admission
16	2001	Frangié et al. [21]	*N. nova*	50, M	Kidney Tx	Tacrolimus Mycophenolate mofetil Corticosteroids	Fever Abdominal pain	Perinephric abscess	Kidney	NR	vancomycin + imipenem	imipenem (2 months) Switch roxithromycin (7 months)	None	Survived after 8 months of follow-up
17	2003	Benes et al. [22]	*N. asteroides*	55, F	None	None	Skin lesions Fever Abdominal pain Fatigue	Skin pustules and ulcer Peritoneal cavity fluid	Skin Kidney Liver Peritoneal cavity Lungs (suspected)	More than 3 months	ampicillin, cefadroxil, roxithromycin gentamicin, cephazolin, oxacillin, clindamycin, ciprofloxacin, rifampicin	TMP-SMX + amikacin Switch cefotaxime + amikacin (9 weeks)	Nephrectomy Surgical drainage	Survived after 3 years of follow-up
18	2003	Qu et al. [23]	*N. asteroides*	37, M	Small bowel Tx	Corticosteroids Tacrolimus Azathioprine	Dysuria Suprapubic pain Scrotal swelling Fever	Urine Blood	Prostate Blood	4 days	ciprofloxacin Then ampicillin-sulbactam	ceftriaxone + TMP-SMX (6 months)	None	Survived at discharge of hospital
19	2005	Routh et al. [24]	*N. asteroides*	78, M	p-ANCA GN	Alkylating agents Corticosteroids	Fatigue Weight loss Fever Scrotal swelling	Testicular abscess Blood	Testis Blood	NR	None	TMP-SMX Then meropenem + tetracycline (6 months)	Orchiectomy	Survived at discharge of hospital
20	2005	Severo et al. [25]	*N.farcinica*	76, M	Chronic low back pain	Corticosteroids	Cough Fever	Thyroid abscess Sputum Blood	Thyroid Lungs Blood Heart Kidney Brain Bones Soft tissue (autopsy)	NR	None	TMP-SMX	None	Deceased on the second day of treatment
21	2006	Kepkep et al. [26]	*Nocardia sp.*	32, F	Pregnancy	None	Fatigue Fever Vomiting Pelvic pain	Tubo-ovarian abscess Appendicitis	Ovary Appendix Lungs (suspected)	40 days	cefazolin	TMP-SMX (6 months)	Salpingo-oophorectomy + appendicectomy	Survived after 6 months of treatment
22	2006	Gallo et al. [27]	*Nocardia sp.*	68, F	Vulvar leiomyosarcoma	NR	Painful lesion in the right hemivulva	Vulvar lesion biopsy	Vulva Bone	NR	None	TMP-SMX (5 months)	Vulvectomy	Survived after 6 months of follow-up
23	2007	Van Luin et al. [41]	*N. farcinica*	52, M	Kidney Tx	Mycophenolate mofetil Tacrolimus Corticosteroids	Abdominal pain Fever	Renal abscess	Kidney	NR	vancomycin	imipenem/cilastatin Then TMP-SMX (6 months)	Transplantectomy	Survived 4 years after discharge of hospital
24	2009	Dehghani et al. [28]	*Nocardia sp*	22, M	T-cell ALL Stem cell Tx	Alkylating agents Cyclosporin Methotrexate Corticosteroids	Fever Hematuria Scrotal swelling Convulsions	Testicular abscess	Testis Brain	NR	ceftriaxone	TMP-SMX (2 weeks)	None	Deceased 3 weeks after discharge of hospital
25	2011	Tanioka et al. [29]	*N. farcinica*	59, F	Myasthenia gravis	Corticosteroids Tacrolimus	Malaise Cough Breath shortness	Sputum	Lungs Kidney Brain Blood	4 days	piperacillin-tazobactam + ciprofloxacin	imipenem-cilastatin + TMP-SMX Then meropenem + amikacin Then linezolid (38 days) Then TMP	None	Survived at discharge of hospital
26	2012	De Montmollin et al. [30]	*N. farcinica*	68, F	Anorexia nervosa	None	Weight loss Fever Dyspnea	Renal abscess	Kidney Lungs Brain	NR	ceftriaxone + erythromycin	TMP-SMX + amikacin + imipenem + ciprofloxacin	None	Deceased 2 months after the start of therapy
27	2013	Yamaguchi et al. [31]	*N. brasiliensis*	77, M	None	None	Skin infection Arthralgia Fever Scrotal swelling	Skin biopsy Testicular abscess	Skin Testis	16 days	acyclovir + cefepime Then vancomycin	meropenem + vancomycin Then TMP-SMX (4 months) + minocycline + imipenem + cilastatin (2 months)	Orchiectomy	Survived at discharge of hospital
28	2015	Poisnel et al. [32]	*N. veterana*	51, M	Glioblastoma Chronic kidney disease	Chemotherapy Corticosteroids	Chest pain Weight loss Dysuria	Urine Blood	Prostate Blood Lungs (suspected)	4 days	TMP-SMX + ceftriaxone	TMP-SMX + ceftriaxone	None	Deceased 2 months after admission
29	2016	Eren et al. [33]	*N. otitidiscaviarum*	69, F	None	None	Hemiparesis Fever	Renal abscess Brain abscess	Kidney Brain	5 days	ceftriaxone + metronidazole	meropenem + amikacin Switch meropenem + TMP-SMX (8 weeks) Then TMP-SMX only (1 year)	Stereotaxic craniotomy	Survived after 1 year of targeted antibiotic
30	2016	Scorey et al. [34]	*N. farcinica*	68, M	Psoriasis and psoriatic arthritis	Corticosteroids Etanercept	Dysuria Abdominal pain Fever	Blood	Prostate Blood	NR	ceftriaxone + gentamicin + flucloxacillin Switch ciprofloxacin	ciprofloxacin + TMP-SMX (8 months)	None	Survived after targeted treatment
31	2017	Bonifaz et al. [35]	*N. brasiliensis*	24, F	NR	NR	Fever Weight loss Fatigue Perineal and loin pain Skin nodules at medial thigh, on the external genital tract and pelvis	Ovarian biopsy	Ovary Perineum Thigh Pelvis	NR	None	amikacin + TMP-SMX + Switch amoxicillin/clavulanate + TMP-SMX	None	Survived after 6 months of follow-up
32	2018	Roy et al. [36]	*N. paucivorans*	63, F	Lung Tx	Mycophenolate mofetil Tacrolimus Corticosteroids	Abdominal pain Nausea Vomiting	Renal abscess	Kidney	NR	None	imipenem + TMP-SMX (8 weeks) Then TMP-SMX (9–12 months)	Nephrectomy	Survived at discharge of hospital
33	2019	Sakamaki et al. [9]	*N. farcinica*	70, M	Interstitial lung disease Diabetes mellitus	Corticosteroids Cyclosporin	Turbid urine	Urine	Prostate	NR	levofloxacin	TMP-SMX Switch imipenem/cilastatin Switch TMP-SMX + amoxicillin/clavulanate (12 months)	None	Survived at discharge of hospital
34	2021	Marques et al. [37]	*Nocardia sp.*	78, M	Heavy alcohol consumption	None	Seizures Fever Cough	Prostate abscess	Prostate Brain Lung Liver Kidney Spleen	NR	amoxicillin-clavulanate Then ceftriaxone + metronidazole	TMP-SMX + amikacin + imipenem (8 months) ceftriaxone + amoxicillin/clavulanate (4 months)	None	Survived after 12 months of follow-up
35	2021	Pan et al. [38]	*N. farcinica*	66, F	Idiopathic membranous nephropathy	Corticosteroids Tacrolimus Cyclophosphamide	Fever Fatigue	EBUS-TBNA biopsy of the pulmonary lesions	Lungs Brain Kidney	3 months	cephalosporin + levofloxacin (3 weeks) Piperacillin-tazobactam (8 days)	meropenem + TMP-SMX (2 weeks) Switch TMP-SMX (6 months)	None	Survived after 6 months of follow-up
36	2022	Shen et al. [39]	*Nocardia sp.*	66, F	Diabetes mellitus Adenomyosis Leiomyoma Chronic cervicitis	None	Abdominal pain Pollakiuria Difficult defecation	Tubo-ovarian abscess	Ovary Uterus Colon Rectum	NR	ceftriaxone + metronidazole + doxycycline Switch to imipenem + doxycycline	imipenem + doxycycline (14 days)	Total hysterectomy and bilateral adnexectomy	Survived at discharge of hospital

Legend: ALL = acute lymphoblastic leukemia; BAL = bronchoalveolar lavage; COPD = chronic obstructive pulmonary disease; CSF = cerebrospinal fluid, EBUS-TBNA = endobronchial ultrasound-guided transbronchial needle aspiration; F = female; HIV = human immunodeficiency virus; M = man; NA = not applicable; NR = not reported; p-ANCA GN = perinuclear antineutrophil cytoplasmic antibody-positive glomerulonephritis; *sp.* = species; Tx = transplantation; TMP-SMX = trimethoprim-sulfamethoxazole

The diagnosis of nocardiosis requires the identification of *Nocardia* in a bacteriological sample. *Nocardia* can be isolated by culture from different samples such as sputum, bronchoalveolar lavage fluid, abscess fluid and blood [4, 7]. Because of the slow-growing nature of *Nocardia*, isolates can take up to 2 weeks to grow on routine culture media used in clinical laboratories, making them difficult to identify [7]. Nevertheless, identification of *Nocardia* species is important because antimicrobial susceptibility varies among species [1, 2, 4–7].

Optimal management for nocardiosis has not been established because of the lack of comparative controlled studies, due to the rarity of the cases. TMP-SMX remains the first-choice agent due to good responses as observed since 1950s and because of its good penetration in the CNS [1, 5, 6]. The main adverse reactions to high-dose TMP-SMX therapy are myelosuppression, hepatotoxicity, renal insufficiency and allergic reaction. Linezolid is a good alternative for disseminated and CNS nocardiosis, but its toxicity includes a high risk of myelosuppression and peripheral neuropathy [1]. Initial multidrug therapy is recommended for most forms of nocardiosis (except limited skin infection). Therapeutic changes should be based on initial therapy, susceptibility results and individual specificities. Treatment duration is generally extended to minimize the risk of disease relapse [6]. *Nocardia* infections may recur because of the slow replication of the pathogen and its intracellular presence [7]. Immunodeficient hosts and/or patients with CNS nocardiosis should receive at least 12 months of antimicrobial therapy (initially intravenous therapy for 4–6 weeks followed by oral agent for 6–12 months) [1, 6, 38]. Neurosurgical drainage should be considered in case of large brain abscess not responding to antimicrobial therapy. Patients with surgical and antibiotics therapy had lower mortality [6].

## Conclusion

To the best of our knowledge, this is the first case of *N. brasiliensis* simultaneously infecting skin, testis, brain and spinal cord in an immunocompromised patient. Our case highlights the difficulty of nocardiosis diagnosis due to complex clinical manifestations. Even though pulmonary, neurological and dermatological involvement are commonly described, the two latter forms may have, as in our patient, tricky clinical presentations and the disease may spread to virtually any organ such as testis.

Knowledge on atypical forms of nocardiosis remains scarce. With our case, we aim to both raise clinician’s awareness and add our experience to the handful of cases described in the literature.

## Data Availability

No datasets were generated or analysed during the current study.

## Abbreviations

CNS: Central Nervous System
CRP: C-reactive Protein
CT: Computed Tomography
MIC: Minimum Inhibitory Concentration
MRI: Magnetic Resonance Imaging
OD: Once a Day
TMP-SMX: Trimethoprim-sulfamethoxazole
